# Supplementary material for: Lateral transduction is inherent to the life cycle of the archetypical Salmonella phage P22
Source: Nat Commun. 2021 Nov 8;12:6510. doi: 10.1038/s41467-021-26520-4 (PMC8575938; doi:10.1038/s41467-021-26520-4)
Supplement: Supplementary file 1 — Supplementary Information [file 41467_2021_26520_MOESM1_ESM.pdf]

Supplementary Tables.

Table S1. Statistical values.

Figure 4a	*p* value
HDR	0.0009
HD+1	< 0.0001
HD+2	< 0.0001
HD+3	0.0003
HD+4	< 0.0001
HD+5	< 0.0001
HD+6	0.0002
HD+7	0.0006
HD+8	0.0006
HD+9	0.0024
HD+10	0.0009
HD+11	0.0029
HD+12	0.0266
**Figure 4b**	***p* value**
HDR	0.0002
HD+1	< 0.0001
HD+2	0.0002
HD+3	0.0001
HD+4	0.0014
HD+5	< 0.0001
HD+6	0.0001
HD+7	0.0023
HD+8	0.0005
HD+9	0.0081
HD+10	0.0041

Figure 5a. Lateral transfer	*p* value
0-30	0.9974
0-60	0.0899
0-90	< 0.0001
0-120	< 0.0001
0-150	< 0.0001
0-180	< 0.0001
0-210	< 0.0001
0-240	< 0.0001
0- -	< 0.0001
**Figure 5b. Phage titre**	***p* value**
0-30	0.9975
0-60	0.9920
0-90	0.9101
0-120	0.1489
0-150	0.0017

0-180	< 0.0001
0-210	< 0.0001
0-240	< 0.0001
0- -	< 0.0001

**Figure S5.<br>qPCR EXCISION**	***p* value**
P22 0-30	>0.9999
P22 0-60	0,1118
P22 0-90	0,0405
P22 ts 0-30	0,1916
P22 ts 0-60	0,6757
P22 ts 0-90	>0.9999
P22 Δ*pri* 0-30	>0.9999
P22 Δ*pri* 0-60	0,7658
P22 Δ*pri* 0-90	0,2902
ES18 0-60	0,6209
ES18 0-90	0,0258
ES18 0-120	0,0033

**Figure S5.<br>qPCR CIRCULARISATION**	**p value**
P22 0-30	>0.9999
P22 0-60	0,5923
P22 0-90	<0.0001
P22 ts 0-30	0,9749
P22 ts 0-60	0,3107
P22 ts 0-90	0,8334
ES18 0-60	0,9989
ES18 0-90	0,0003
ES18 0-120	<0.0001

**Figure S7**	***p* value**
HD+1	0.1760
HD+2	0.2911
HD+3	0.0741
HD+4	0.8390
HD+5	0.1275
HD+6	0.4479
HD+7	0.5348
HD+8	0.0677
HD+9	0.6282
HD+10	0.9924
HD+11	0.1713
HD+12	0.7911

Figure S8a	*p* value
HD+1	0.0207
HD+2	0.0131
**Figure S8b**	***p* value**
HD+1	0.0013
HD+2	0.0009

Figure S9	*p* value
HD+1	0.0008
HD+2	0.0023
HD+4	0.0082
tetM^G	0.9364

Figure S10. P22 LT	*p* value
wt-Δ*int*	0.0003
wt-Δ*xis*	0.0002
wt-Δ*int-xis*	0.0002
**Figure S10. P22 titre**	***p* value**
wt-Δ*int*	< 0.0001
wt-Δ*xis*	< 0.0001
wt-Δ*int-xis*	< 0.0001

Figure S10. ES18 LT	*p* value
wt-Δ*int*	0.0136
wt-Δ*xis*	0.0335
wt-Δ*int-xis*	0.0434
**Figure S10. ES18 titre**	***p* value**
wt-Δ*int*	< 0.0001
wt-Δ*xis*	< 0.0001
wt-Δ*int-xis*	< 0.0001

Table S2. Strains used in this study.

Strain	Description	Reference
E. coli DC10B	K-12-derivative cloning strain; *dam*$^+$ *Ddcm DhsdRMS endA1 recA1*	[1]
VE18590	*E. faecalis* V583 derivative. Non-lysogenic, EfCIV583-negative.	[2]
VE18562	*E. faecalis* V583 derivative. fp1 lysogen, EfCIV583-negative.	[2]
JP18938	LT2 ΔFels-1 ΔGifsy-2 ΔGifsy-1 ΔFels-2	This work
JP22117	SV1208 ΔFels-1	This work
JP18983	JP18938 P22 lysogen	This work
JP18985	JP18938 ES18 lysogen	This work
JP22119	JP22117 P22 tsc_2*29* lysogen	This work
JP20460	JP18938 Δ*rfb*P-*rfb*B::*Km*R	This work
JP20590	JP18938 P22 lysogen Δ*int*	This work
JP20591	JP18938 P22 lysogen Δ*xis*	This work
JP20592	JP18938 P22 lysogen Δ*int-xis*	This work
JP20593	JP18938 P22 lysogen Δ*orf12*	This work
JP20595	JP18938 ES18 lysogen Δ*int*	This work
JP20596	JP18938 ES18 lysogen Δ*xis*	This work
JP20597	JP18938 ES18 lysogen Δ*int-xis*	This work
JP19020	JP18938 Δ*att*B P22	This work
JP22118	JP22117 Δ*att*B P22	This work
JP19086	JP19020 *gpt*ΩTetA (6 kb upstream P22 *att*B)	This work
JP19087	JP19020 STM0331ΩTetA (6 kb downstream P22 *att*B, HF1)	This work
JP19088	JP19020 *prp*RΩTetA (47 kb downstream P22 *att*B, HF2)	This work
JP19089	JP19020 *mal*ZΩTetA (88 kb downstream P22 *att*B, HF3)	This work
JP19090	JP19020 *yaj*GΩTetA (130 kb downstream P22 *att*B, HF4)	This work
JP19091	JP19020 *yba*NΩTetA (170 kb downstream P22 *att*B, HF5)	This work
JP19092	JP19020 *ybb*VΩTetA (213 kb downstream P22 *att*B, HF6)	This work
JP19093	JP19020 STM0557ΩTetA (246 kb downstream P22 *att*B, HF7)	This work
JP19094	JP19020 *ahp*FΩTetA (304 kb downstream P22 *att*B, HF8)	This work
JP19095	JP19020 *ybe*LΩTetA (348 kb downstream P22 *att*B, HF9)	This work
JP19096	JP19020 STM0699ΩTetA (392 kb downstream P22 *att*B, HF10)	This work
JP19097	JP19020 *nei*ΩTetA (425 kb downstream P22 *att*B, HF11)	This work
JP19098	JP19020 *mod*FΩTetA (474 kb downstream P22 *att*B, HF12)	This work
JP20469	JP18983 *gpt*ΩTetA (6 kb upstream P22 *att*B)	This work
JP20470	JP18983 STM0331ΩTetA (6 kb downstream P22 *att*B, HF1)	This work
JP20471	JP18983 *prp*RΩTetA (47 kb downstream P22 *att*B, HF2)	This work
JP20472	JP18983 *mal*ZΩTetA (88 kb downstream P22 *att*B, HF3)	This work
JP20473	JP18983 *yaj*GΩTetA (130 kb downstream P22 *att*B, HF4)	This work
JP20474	JP18983 *yba*NΩTetA (170 kb downstream P22 *att*B, HF5)	This work
JP20475	JP18983 *ybb*VΩTetA (213 kb downstream P22 *att*B, HF6)	This work
JP20476	JP18983 STM0557ΩTetA (246 kb downstream P22 *att*B, HF7)	This work
JP20477	JP18983 *ahp*FΩTetA (304 kb downstream P22 *att*B, HF8)	This work
JP20478	JP18983 *ybe*LΩTetA (348 kb downstream P22 *att*B, HF9)	This work
JP20479	JP18983 STM0699ΩTetA (392 kb downstream P22 *att*B, HF10)	This work
JP20480	JP18983 *nei*ΩTetA (425 kb downstream P22 *att*B, HF11)	This work
JP20481	JP18983 *mod*FΩTetA (474 kb downstream P22 *att*B, HF12)	This work
JP21928	JP22118 *gpt*ΩTetA (6 kb downstream P22 *att*B)	This work
JP21929	JP22118 *prp*RΩTetA (47 kb downstream P22 *att*B)	This work
JP21922	JP22119 *gpt*ΩTetA (6 kb downstream P22 *att*B)	This work
JP21923	JP22119 *prp*RΩTetA (47 kb downstream P22 *att*B)	This work
JP20482	JP18985 *gpt*ΩTetA (6 kb upstream P22 *att*B)	This work
JP20483	JP18985 STM0331ΩTetA (6 kb downstream P22 *att*B, HF1)	This work
JP20484	JP18985 *prp*RΩTetA (47 kb downstream P22 *att*B, HF2)	This work
JP20485	JP18985 *mal*ZΩTetA (88 kb downstream P22 *att*B, HF3)	This work
JP20487	JP18985 *yba*NΩTetA (170 kb downstream P22 *att*B, HF4)	This work
JP20488	JP18985 *ybb*VΩTetA (213 kb downstream P22 *att*B, HF5)	This work
JP20489	JP18985 STM0557ΩTetA (246 kb downstream P22 *att*B, HF6)	This work
JP20490	JP18985 *ahp*FΩTetA (304 kb downstream P22 *att*B, HF7)	This work
JP20491	JP18985 *ybe*LΩTetA (348 kb downstream P22 *att*B, HF8)	This work
JP20493	JP18985 *nei*ΩTetA (425 kb downstream P22 *att*B, HF9)	This work
JP20494	JP18985 *mod*FΩTetA (474 kb downstream P22 *att*B, HF10)	This work
JP19438	JP20470 Δ*rfb*P-*rfb*B::*Km*R	This work
JP19439	JP20471 Δ*rfb*P-*rfb*B::*Km*R	This work
JP19440	JP20472 Δ*rfb*P-*rfb*B::*Km*R	This work
JP19441	JP20473 Δ*rfb*P-*rfb*B::*Km*R	This work

JP19442	JP20474 Δ*rfb*P-*rfb*B::*Km*R	This work
JP19443	JP20475 Δ*rfb*P-*rfb*B::*Km*R	This work
JP19444	JP20476 Δ*rfb*P-*rfb*B::*Km*R	This work
JP19445	JP20477 Δ*rfb*P-*rfb*B::*Km*R	This work
JP19446	JP20478 Δ*rfb*P-*rfb*B::*Km*R	This work
JP19447	JP20479 Δ*rfb*P-*rfb*B::*Km*R	This work
JP19448	JP20480 Δ*rfb*P-*rfb*B::*Km*R	This work
JP19449	JP20481 Δ*rfb*P-*rfb*B::*Km*R	This work
JP21422	JP19020 Cat marker 6 kb downstream P22 *att*B + TetA marker 6 kb upstream P22 *att*B	This work
JP21423	JP19020 Cat marker 6 kb downstream P22 *att*B + TetA marker 10 kb up downstream P22 *att*B	This work
JP21424	JP19020 Cat marker 6 kb downstream P22 *att*B + TetA marker 29 kb up downstream P22 *att*B	This work
JP21425	JP19020 Cat marker 6 kb downstream P22 *att*B + TetA marker 32 kb up downstream P22 *att*B	This work
JP21426	JP19020 Cat marker 10 kb downstream P22 *att*B + TetA marker 29 kb upstream P22 *att*B	This work
JP21427	JP19020 Cat marker 10 kb downstream P22 *att*B + TetA marker 32 kb upstream P22 *att*B	This work
JP21428	JP19020 Cat marker 15.6 kb downstream P22 *att*B + TetA marker 29 kb upstream P22 *att*B	This work
JP21429	JP19020 Cat marker 15.6 kb downstream P22 *att*B + TetA marker 32 kb upstream P22 *att*B	This work
JP21430	JP19020 Cat marker 29 kb downstream P22 *att*B + TetA marker 32 kb upstream P22 *att*B	This work
JP21431	JP18983 Cat marker 6 kb downstream P22 *att*B + TetA marker 6 kb upstream P22 *att*B	This work
JP21432	JP18983 Cat marker 6 kb downstream P22 *att*B + TetA marker 10 kb up downstream P22 *att*B	This work
JP21433	JP18983 Cat marker 6 kb downstream P22 *att*B + TetA marker 29 kb up downstream P22 *att*B	This work
JP21434	JP18983 Cat marker 6 kb downstream P22 *att*B + TetA marker 32 kb up downstream P22 *att*B	This work
JP21435	JP18983 Cat marker 10 kb downstream P22 *att*B + TetA marker 29 kb upstream P22 *att*B	This work
JP21436	JP18983 Cat marker 10 kb downstream P22 *att*B + TetA marker 32 kb upstream P22 *att*B	This work
JP21437	JP18983 Cat marker 15.6 kb downstream P22 *att*B + TetA marker 29 kb upstream P22 *att*B	This work
JP21438	JP18983 Cat marker 15.6 kb downstream P22 *att*B + TetA marker 32 kb upstream P22 *att*B	This work
JP21439	JP18983 Cat marker 29 kb downstream P22 *att*B + TetA marker 32 kb upstream P22 *att*B	This work
JP20600	JP20590 *prp*RΩTetA (47 kb downstream P22 *att*B, HF2)	This work
JP20601	JP20591 *prp*RΩTetA (47 kb downstream P22 *att*B, HF2)	This work
JP20602	JP20592 *prp*RΩTetA (47 kb downstream P22 *att*B, HF2)	This work
JP20605	JP20595 *prp*RΩTetA (47 kb downstream P22 *att*B, HF2)	This work
JP20606	JP20596 *prp*RΩTetA (47 kb downstream P22 *att*B, HF2)	This work
JP20607	JP20597 *prp*RΩTetA (47 kb downstream P22 *att*B, HF2)	This work
JP20757	JP20602 pJP2534	This work
JP21704	*E. coli* DC10B pJP2563	This work
JP21747	*E. coli* DC10B pJP2564	This work
JP21748	*E. coli* DC10B pJP2565	This work
JP21896	*E. coli* DC10B pJP2566	This work
JP21706	*E. coli* DC10B pJP2568	This work
JP21767	VE18590 *celA*Ω*tet*M (12.3 kb upstream of pp1 *att*B)	This work
JP21855	VE18590 EF0370Ω*tetM* (12.3 kb downstream of pp1 *att*B, HF1)	This work
JP22112	VE18590 EF0394Ω*tetM* (40.7 kb downstream of pp1 *att*B, HF2)	This work
JP22021	VE18590 EF0480Ω*tetM* (121.1 kb downstream of pp1 *att*B, HF4)	This work
JP21771	VE18590 EF2276Ω*tetM* (1.87 Mb downstream of pp1 *att*B)	This work
JP21768	VE18562 *celA*Ω*tetM* (12.3 kb upstream of pp1 *att*B)	This work
JP21856	VE18562 EF0370Ω*tetM* (12.3 kb downstream of pp1 *att*B, HF1)	This work
JP21857	VE18562 EF0394Ω*tetM* (40.7 kb downstream of pp1 *att*B, HF2)	This work
JP22110	VE18562 EF0480Ω*tetM* (121.1 kb downstream of pp1 *att*B, HF4)	This work

JP21772	VE18562 EF2276Ω*tetM* (1.87 Mb downstream of pp1 *att*B)	This work

Table S3. Oligonucleotides used in this study.

Mutagenesis	Primers	Sequence (5’-3’)
**LT2 ΔFels-1**	**LT2-AFels1-1m**	GGGGCACTCCTGGGGCAGTAGATGCCAGTTGTTGATTCAG<br>TATATCTACTGTGTAGGCTGGAGCTGCTTCG
	**LT2-AFels1-2c**	GGCATCATACTGTACACTGTCATATGCCATATATTTAAACGC<br>TAAAAGGGCATATGAATATCCTCCTTA
**LT2 ΔGifsy-2**	**LT2-AGifsy2-13m**	CGCTGGAGTATACCTTGTTTAGCGATTTATTGAACCCCGATC<br>ACACCTGTGTAGGCTGGAGCTGCTTCG
	**LT2-AGifsy2-14c**	GCAAATCGCGCTACGCAGAATGTTCATCTTTTCAGGCACAA<br>ACGGCCGGTCCATATGAATATCCTCCTTAG
**LT2 ΔGifsy-1**	**LT2-AGifsy1-1m**	CAAGTAACGAGGCGACATCAAACCTTGAGTTATTAAATAAAT<br>GGAGAATAGTGTGTAGGCTGGAGCTGCTTCG
	**LT2-AGifsy1-2c**	GTCCCTGATATGGGCGTGCAGGGTCGGTGAACTCCGTCAG<br>GCTGAAGCATATGAATATCCTCCTTA
**LT2 ΔFels-2**	**LT2-AFels2-1m**	CTTCAAGCTCCATGCTGGTCGTAAATCCATTATCCGGGCTG<br>ACAGAATGCTGTGTAGGCTGGAGCTGCTTCG
	**LT2-AFels2-2c**	TTCCTTGAGCATCTTCGGGCGGATCTCGGTAACGGGTGTGT<br>TACCCAGAGCATATGAATATCCTCCTTA
**LT2 Δ*att*B P22**	**LT2-AattB-1m**	CCTTCTACCCCGTGATTCACCCGCGTGAACACACCCTTCTC<br>ATGTGTAGGCTGGAGCTGCTTCG
	**LT2-AattB-2c**	GGATACTGCTTATGTTTTGCTAGTTGTGTACCCGAGAGTGTA<br>CCCGGTCCATATGAATATCCTCCTTAG
**LT2 Δ*rfb*P-*rfb*B P22**	**LT2-ArfbP-1m**	TTTTACGCAGGCTAATTTATACAATTATTATTCAGTACTTCTC<br>GGTAAGCTTGTGTAGGCTGGAGCTGCTTCG
	**LT2-ArfbB-2c**	TTTATTGGCAAATTAAATACCACATTAAATACGCCTTATGGA<br>ATAGAAAACATATGAATATCCTCCTTA
***tet*A marker 6 kb upstream P22 *att*B**	**LT2-tetA-11m**	TCAGACACGATAAGTCTCCTTGGCGGTGGTCTGAAAAACGT<br>TCTTACAGGCCACAGGGTTCGCTGTTAATCACTTTACTT
	**LT2-tetA-12c**	ACCCGCAGGCGGAGAAAGTGGTATTCTCAGTCGCATCTCGT<br>AAGAGTTATCAATTCCGCTGGTTATCAAGAGGGTCATTA
***tet*A marker 6 kb downstream P22 *att*B**	**LT2-tetA-3m**	GGTTGCGCATTATTCACGGCGGCCGTGGCTAAAACGCCCT<br>GCATCCCCGCGCGGTTAAGGCGCTGTTAATCACTTTACTT
	**LT2-tetA-4c**	AATGCCGTAGCGTTTGCCACCGTTCACAAGATATTGAACCA<br>GTTTCATTGCAGTCTTCTTGGTTATCAAGAGGGTCATTA
***tet*A marker 47 kb downstream P22 *att*B**	**LT2-tetA-6m**	ATTGCATCATAAAAATACCCGCCTGGGTTTCCGGCGGGTAT<br>ATTTATTCCTGGCAACCGGCGCTGTTAATCACTTTACTT
	**LT2-tetA-7c**	GCGTCCTGAATGCCTGATGGCGCTACGTTTACTGCAGGCC<br>GTCATCCGGCAAAACGGATGGGTTATCAAGAGGGTCATTA
***tet*A marker 88 kb downstream P22 *att*B**	**LT2-tetA-9m**	GCGAGGGAAGACTTAGTTTACCCGCGATTTCCGCGACGGT<br>ATGGATGAATCGTTGAGGCGCGCTGTTAATCACTTTACTT
	**LT2-tetA-10c**	CATTGATAAAAGGCTCGCGAATGCGAGCCTTTTTTATGCGT<br>AAGGTGTTACGAACCACCTGGTTATCAAGAGGGTCATTA
***tet*A marker 130 kb downstream P22 *att*B**	**LT2-tetA-13m**	CGTAAATATTGACTTGACATAGGCATCTACAGACCCGGCTT<br>CTGCCGGGTCTTGTTGTTGCGCTGTTAATCACTTTACTT
	**LT2-tetA-14c**	GCTGATATGTCTCAGGACACCAGTATTCACGATTTTATCAAG<br>CAAAACGCCCGTTAATAGGGTTATCAAGAGGGTCATTA
***tet*A marker 170 kb downstream P22 *att*B**	**LT2-tetA-15m**	CTGCTGATTTTTATGTGGCGGATACCGGTGATTGATGAAAA<br>GCAACAAAAGCGCTGAAGCCGCTGTTAATCACTTTACTT
	**LT2-tetA-16c**	TGTGCTCGAAAACGGTCGAATTTACTGGCTGTGAACGACAA<br>TTGCAACAGCGATTCGTGTGGTTATCAAGAGGGTCATTA
***tet*A marker 213 kb downstream P22 *att*B**	**LT2-tetA-17m**	TCACTTTATATAAAAAGAAAGCAACGCAACGTATTGCTTCAG<br>CTTAAAAATAATTATATCCGCTGTTAATCACTTTACTT
	**LT2-tetA-18c**	GATAACGTTGTTATGGGTTTAGCTATGAGGAACAAATAAATA<br>TAAAATAAAGAATTAGTAGGTTATCAAGAGGGTCATTA
***tet*A marker 246 kb**	**LT2-tetA-19m**	TGTAGGAATCCCCGCCGCCCGTTACCCATTGGTGGCGGGG<br>AACATTAATTATACATGAATCGCTGTTAATCACTTTACTT

**downstream P22 *att*B**	**LT2-tetA-20c**	CATGGGACGTTAGAACAGGGGCGAGGACAAATGAGAATATTACGGAAATAATTAAATAACGGTTATCAAGAGGGTCATTA
***tet*A marker 304 kb**	**LT2-tetA-25m**	GCCTTTGATTATCTGATTCGCACCAAAATCGCATAAAAGAAGTAAGCACACCTGCAAGGCGCTGTTAATCACTTTACTT
**downstream P22 *att*B**	**LT2-tetA-26c**	AAACACCGCAGGCCCGAATAGCTTACACTATCGGGCCATTTACGATGGCCAGTTAACTGGGGTTATCAAGAGGGTCATTA
***tet*A marker 348 kb**	**LT2-tetA-27m**	GTGTGGCCACGATCAGTTCCAGCGCAGGCCGTTTGAGCCGTAAGGTTCATTATCGTGAGCCGCTGTTAATCACTTTACTT
**downstream P22 *att*B**	**LT2-tetA-28c**	GAAGAGATCCGCAAACTGGAAGCCCAACTGCACGCTTAACATGGCGGACGGCAACCGTCCGGTTATCAAGAGGGTCATTA
***tet*A marker 392 kb**	**LT2-tetA-21m**	TGAGAAAGAAGCCGTTGAAATCGTTAGCGAAGTATTGAAAAACGCCTGATGGGCGATATGCGCTGTTAATCACTTTACTT
**downstream P22 *att*B**	**LT2-tetA-22c**	AGGCCTGATAAGCGCAGCGCCGTTAATACAAAAAAGGAGCCGTAAGGCTCCTTTTTCTTCGGTTATCAAGAGGGTCATTA
***tet*A marker 425 kb**	**LT2-tetA-29m**	CGCATTGCCAGAAATAGCCGGAACCGACATCGGCGAGCGGCTATTGCCTGATGGCGCGACCGCTGTTAATCACTTTACTT
**downstream P22 *att*B**	**LT2-tetA-30c**	AGCAGTGGCCCGTTTTATATCAACCTATGCGCCCAAACAACCTTTGTAGGGCTGATAAGCGGTTATCAAGAGGGTCATTA
***tet*A marker 474 kb**	**LT2-tetA-23m**	TATCGACCGTTATTATTATTCTTAATAAAAGGAGAGTGGTTCCAGAATGGCGCGCGGCGCCGCTGTTAATCACTTTACTT
**downstream P22 *att*B**	**LT2-tetA-24c**	AGGGCGAACGTTATAAGTACGTTTCCGGACGATGCAATAATTAAATGTATTATCAGAATGGGTTATCAAGAGGGTCATTA
***cat* marker 6 kb**	**LT2-STM0331-cat-1m**	GGTTGCGCATTATTCACGGCGGCCGTGGCTAAAACGCCCTGCATCCCCGCGCGGTTAAGGGGCGCGCCTACCTGTGACGG
**downstream P22 *att*B**	**LT2-STM0331-cat-2c**	AATGCCGTAGCGTTTGCCACCGTTCACAAGATATTGAACCAGTTTCATTGCAGTCTTCTTGGAATAGGAACTTCATTTAAATGGC
***tet*A marker 10 kb**	**LT2-stbE-tetA-2c**	CTCCTGAGTTTTGATGGGAAATATTCAGGGATACGATCGTATCCCTGAGGGGTTATCAAGAGGGTCATTA
**downstream P22 *att*B**	**LT2-stbE-tetA-1m**	ATCAGCTTAATGCTATTTTTGACAATGGTACATGCTGAGTCTGGCTTGCGCTGTTAATCACTTTACTT
***tet*A marker 29 kb**	**LT2-STM0352-tetA-1m**	CGTTTCCCTCTTTACCGCAGCGTGTCGGCCATTCCGCAACGCTGCCGCAGCGCTGTTAATCACTTTACTT
**downstream P22 *att*B**	**LT2-STM0352-tetA-2c**	CCCGTCAGGAACGCTTGACCTTTCCTTCGTTGTAACGCCTAGCCTTTGCCGGTTATCAAGAGGGTCATTA
***tet*A marker 32 kb**	**LT2-STM0355-tetA-1m**	CCGGTTTCCCGCCGCGCGAGAGGTAATCGTCAATGGCGATCGGTCTGGCGCGCTGTTAATCACTTTACTT
**downstream P22 *att*B**	**LT2-STM0355-tetA-2c**	CTCACGCAAAGAAAAGCGAAGCGTGGCTAGCGTATCGCGACCGGCCTGTGGTTATCAAGAGGGTCATTA
***cat* marker 10 kb**	**LT2-stbE-cat-1m**	ATCAGCTTAATGCTATTTTTGACAATGGTACATGCTGAGTCTGGCTTGGGCGCGCCTACCTGTGACGG
**downstream P22 *att*B**	**LT2-stbE-cat-2c**	CTCCTGAGTTTTGATGGGAAATATTCAGGGATACGATCGTATCCCTGAGGGGAATAGGAACTTCATTTAAATGGC
***cat* marker 15.6 kb**	**LT2-stbB-cat-1m**	CAACTCCCTAGCGATTGAAAATGGTCTGGCGTTTCAACGCCAGACCGTAGGGCGCGCCTACCTGTGACGG
**downstream P22 *att*B**	**LT2-stbB-cat-2c**	GGGACAGTCGCTGCCACCCTTCAATACGCCGTTTCGTATAAATAATTGTCGGAATAGGAACTTCATTTAAATGGC
***cat* marker 29 kb**	**LT2-STM0352-cat-1m**	CGTTTCCCTCTTTACCGCAGCGTGTCGGCCATTCCGCAACGCTGCCGCAGGGCGCGCCTACCTGTGACGG
**downstream P22 *att*B**	**LT2-STM0352-cat-2c**	CCCGTCAGGAACGCTTGACCTTTCCTTCGTTGTAACGCCTAGCCTTTGCCGGAATAGGAACTTCATTTAAATGGC
**P22 Δ*int* (same**	**P22-Aint-3m**	CCCGCTCGTTTTAATGCTGCCCTCCATGCAGTATTAGCGTCATAGTGTGTAGGCTGGAGCTGCTTCG
**primers used for ES18 Δ*int)***	**P22-Aint-4c**	GAGGAGAAGGCGCATAAGAAGTCGCTGGATGATGACAAGAGTCGGGGTCCATATGAATATCCTCCTTAG
**P22 Δ*xis***	**P22-Axis-1m**	ATGTCATCACCCGCGCTCACCTGGACAGTATGCAGCGGAGATTGAAGTGCTGTGTAGGCTGGAGCTGCTTCG

	**P22-Axis-2c**	GAAACAGCGGAGTAAACATGGAATCACACAGCCTCACACTT<br>GATGAGGCCGGTCCATATGAATATCCTCCTTAG
**P22 Δ*int-xis***	**P22-Aint-3m**	CCCGCTCGTTTTAATGCTGCCCTCCATGCAGTATTAGCGTC<br>ATAGTGTGTAGGCTGGAGCTGCTTCG
	**P22-Axis-2c**	GAAACAGCGGAGTAAACATGGAATCACACAGCCTCACACTT<br>GATGAGGCCGGTCCATATGAATATCCTCCTTAG
**P22 Δ*orf12***	**P22-Aorf12-1m**	GGTGGCTTGCTGATTGGCGGATTAACACCAACCGCCAGTG<br>ACGTTCTGGCTGTGTAGGCTGGAGCTGCTTCG
	**P22-Aorf12-2c**	GCGGCTTCCTGATGGATGACAGGCGCTTTACAAGCTCGTCC<br>ATCGCTCTGGGTCCATATGAATATCCTCCTTAG
**ES18 Δ*xis***	**ES18-Axis-1m**	TATGCCATCACCCGCGCTCACGGCGACAGTATGCATCGGA<br>GACTGAAGAGTGTGTAGGCTGGAGCTGCTTCG
	**ES18-Axis-2c**	AGCAATAACAATCCTCGCACTCGCGGGGATTTCTTTTATCC<br>GGAGTAACCGGTCCATATGAATATCCTCCTTAG
**ES18 Δ*int-xis***	**P22-Aint-3m**	CCCGCTCGTTTTAATGCTGCCCTCCATGCAGTATTAGCGTC<br>ATAGTGTGTAGGCTGGAGCTGCTTCG
	**ES18-Axis-2c**	AGCAATAACAATCCTCGCACTCGCGGGGATTTCTTTTATCC<br>GGAGTAACCGGTCCATATGAATATCCTCCTTAG

**Excision**	**Primers**	**Sequence (5'-3')**
**P22 and ES18**	**LT2-attB-1m**	TCAGCACGCAGAAATTACATG
	**LT2-attB-2c**	ACGGAATCATTAACATGATGG

**Circularisation**	**Primers**	**Sequence (5'-3')**
**P22**	**P22-gtrA-4m**	GTTTTGATCGATACAAGCGATC
	**P22-int-7c**	CCTGACTGAACATGCTCGAC
**ES18**	**ES18-orf33-1m**	TCATCAACAGTGCGACAGATG
	**P22-int-7c**	CCTGACTGAACATGCTCGAC

**Housekeeping**	**Primers**	**Sequence (5'-3')**
**P22 and ES18**	**LT2-rapA-1m**	CGCTGAGCTGGCGGTTGAAGG
	**LT2-rapA-2c**	TGCCGCGAGCAACATGAAGCG

Plasmid	Primers	Sequence (5’-3’)	Comment
**pBAD18**			
**pJP2534**	**P22-int-5mS**	ACGCGTCGACCAAATACTTACGTATTATTCGTGCC	
	**P22-xis-4cE**	CCGGAATTCATTCTACGACATCGCTAACGC	
**pBT2bgal**			
**pJP2563**	**pp1-HF-1_FB**	AGTAGGATCCCAAAAGTAGGGGCCTTTTCTGCACCTTTTACAGC	*celA* left flank
	**pp1-tetM_HF-1_R**	CTCAAATTGCGAGATTTGGGTTGCCTTTGTTTCTTGATCAAATTTGAACAAAGGACTGAAACGATATGTCAATTTTGAAAAATG	
	**tetM-F**	GATCAAGAAACAAAGGCAACCCAAATCTCGCAATTTGAG	*tetM* cassette
	**tetM-RS**	GTCAGTCGACGATCTTGTATCATTACTCCATGTATCTATTGATG	
	**pp1-HF-1_FB**	AGTAGGATCCCAAAAGTAGGGGCCTTTTCTGCACCTTTTACAGC	*celA* left flank-*tetM* fusion
	**tetM-RS**	GTCAGTCGACGATCTTGTATCATTACTCCATGTATCTATTGATG	
	**pp1-HF-1_FS**	CATTTTTCAAAATTGACATATCGTTTCAGTCCTTTGTTCAAATTTGTCGACTTATACGTTTTGTGCTTCGACTTTTTCAAGGGTTTCTTC	*celA* right flank
	**pp1-HF-1_RH**	TGCAAAGCTTGTTTTCATTTTGTTTATGTCATCTGAAGTCAAAGATGCG	
**pJP2564**	**pp1-HF1_FB**	ATGAGGATCCGGCTACAACCAATTATTAGCTGACGATTATGTG	EF0370 left flank
	**pp1-tetM_HF1-R**	CTCAAATTGCGAGATTTGGGTTGCCTTTGTTTCTTGATCTTAAATTGCTGTTAAATTAGGGTTATTGATATTTTTAAAATAAAAATC	
	**tetM-F**	GATCAAGAAACAAAGGCAACCCAAATCTCGCAATTTGAG	*tetM* cassette
	**tetM-RS**	GTCAGTCGACGATCTTGTATCATTACTCCATGTATCTATTGATG	
	**pp1-HF1_FB**	ATGAGGATCCGGCTACAACCAATTATTAGCTGACGATTATGTG	EF0370 left flank-*tetM* fusion
	**tetM-RS**	GTCAGTCGACGATCTTGTATCATTACTCCATGTATCTATTGATG	
	**pp1-HF1_FX**	AAATATCAATAACCCTAATTTAACAGCAATTTAATCTAGATTAAACAATCACTAAACAGAGGCTTGTCGACAG	EF0370 right flank
	**pp1-HF1_RNh**	GTCTGCTAGCTGGCGCAAAATGTTCATTATCTTGTGAAAAAAC	
**pJP2565**	**pp1-HF2_FB**	TGCAGGATCCACAACAATGCTAGATGCAGTTTTAGATGCGGACTC	EF0370 left flank
	**pp1-tetM_HF2-R**	CTCAAATTGCGAGATTTGGGTTGCCTTTGTTTCTTGATCTTAGGCTGAGTGTCCTACGATTGTGCTACCTGAGTAACC	
	**tetM-F**	GATCAAGAAACAAAGGCAACCCAAATCTCGCAATTTGAG	*tetM* cassette
	**tetM-RS**	GTCAGTCGACGATCTTGTATCATTACTCCATGTATCTATTGATG	
	**pp1-HF2_FB**	TGCAGGATCCACAACAATGCTAGATGCAGTTTTAGATGCGGACTC	EF0370 left flank-*tetM* fusion
	**tetM-RS**	GTCAGTCGACGATCTTGTATCATTACTCCATGTATCTATTGATG	

	**pp1-HF2_FS**	CTCAGGTAGCACAATCGTAGGACACTCAGCCTAAGTCGACTCAGCAATGATAAAAATAGACGTAAGCAGCTTC	EF0370 right flank
	**pp1-HF2_RNh**	ATGTGCTAGCATCATCAAATTGCAAATAACGGCAACCTCTCTC	
**pJP2566**	**pp1-HF4_FB**	ATGTGGATCCCATTTTAGTTAAATCAGTAATTTTCAATATCGAGGG	EF0480 left flank
	**pp1-HF4_RS**	GAAGCTATCTTAAAATTTAAAATTAACTAAGTCGACCTAAAAAAAGCCCCTTTCCACTTTTTCTG	
	**tetM-FS**	GTACGTCGACGATCAAGAAACAAAGGCAACCCAAATCTCGCAATTTGAG	*tetM* cassette
	**tetM-RS**	GTCAGTCGACGATCTTGTATCATTACTCCATGTATCTATTGATG	
	**pp1-HF4_FS**	CAGAAAAAGTGGAAAGGGGCTTTTTTTAGGTCGACTTAGTTAATTTTAAATTTTAAGATAGCTTC	EF0480 right flank
	**pp1-HF4_RH**	GTCTAAGCTTCTGATTAAAACTTTATTATGATGTCATAGTGAAG	
**pJP2568**	**2196839_FB**	ATGTGGATCCGAAATAAGCAATAATCATTGGTCAACGCCTCTC	EF2276 left flank
	**2196839_RS**	TAGATTATCGAATATTTTCGAAATTTAATTGAATGACAAGTCGACTCAAAGTACGAGGCGATACATTGCTGATAATTTTTTAGAGTATTG	
	**tetM-F**	GATCAAGAAACAAAGGCAACCCAAATCTCGCAATTTGAG	*tetM* cassette
	**tetM-RS**	GTCAGTCGACGATCTTGTATCATTACTCCATGTATCTATTGATG	
	**2196839_FB**	ATGTGGATCCGAAATAAGCAATAATCATTGGTCAACGCCTCTC	EF2276 left flank-*tetM* fusion
	**tetM-RS**	GTCAGTCGACGATCTTGTATCATTACTCCATGTATCTATTGATG	
	**2196839_FSa**	TGCAGTCGACTTGTCATTCAATTAAATTTCGAAAATATTCGATAATCTA	EF2276 right flank
	**2196839_RNh**	GTCAGCTAGCTCAGGTGTAGAAAATTTTACTGTAGATTTTGCC	

Table S4. Plasmids used in this study.

Plasmid	Description	Reference
**pKD46**	Amp^R. Thermosensitive plasmid with Red lambda system	[3]
**pCP20**	Amp^R. Thermosensitive plasmid with FLP recombinase	[3]
**pBAD18**	Amp^R. Expression vector	[4]
**pBT2βgal**	Amp^R, GN; Cm^R, GP. Vector for allelic replacement	[5]
**pRN6680**	pBluescriptΩ2.9-kb pMVN6 *Sma*I-*Hind*II [*tetA(M)*]; Amp^R, Tet^R	[6]
**pJP2534**	pBAD18 *int-xis* P22	This work
**pJP2563**	pBT2βgal with *tetM* from pRN6680 positioned between *E. faecalis* V583 flanking sequences at *celA*; Cm^R, Tet^R	This work
**pJP2564**	pBT2βgal with *tetM* from pRN6680 positioned between *E. faecalis* V583 flanking sequences at *EF0370*; Cm^R, Tet^R	This work
**pJP2565**	pBT2βgal with *tetM* from pRN6680 positioned between *E. faecalis* V583 flanking sequences at *EF0394*; Cm^R, Tet^R	This work
**pJP2566**	pBT2βgal with *tetM* from pRN6680 positioned between *E. faecalis* V583 flanking sequences at *EF0480*; Cm^R, Tet^R	This work
**pJP2568**	pBT2βgal with *tetM* from pRN6680 positioned between *E. faecalis* V583 flanking sequences at *EF2276*; Cm^R, Tet^R	This work

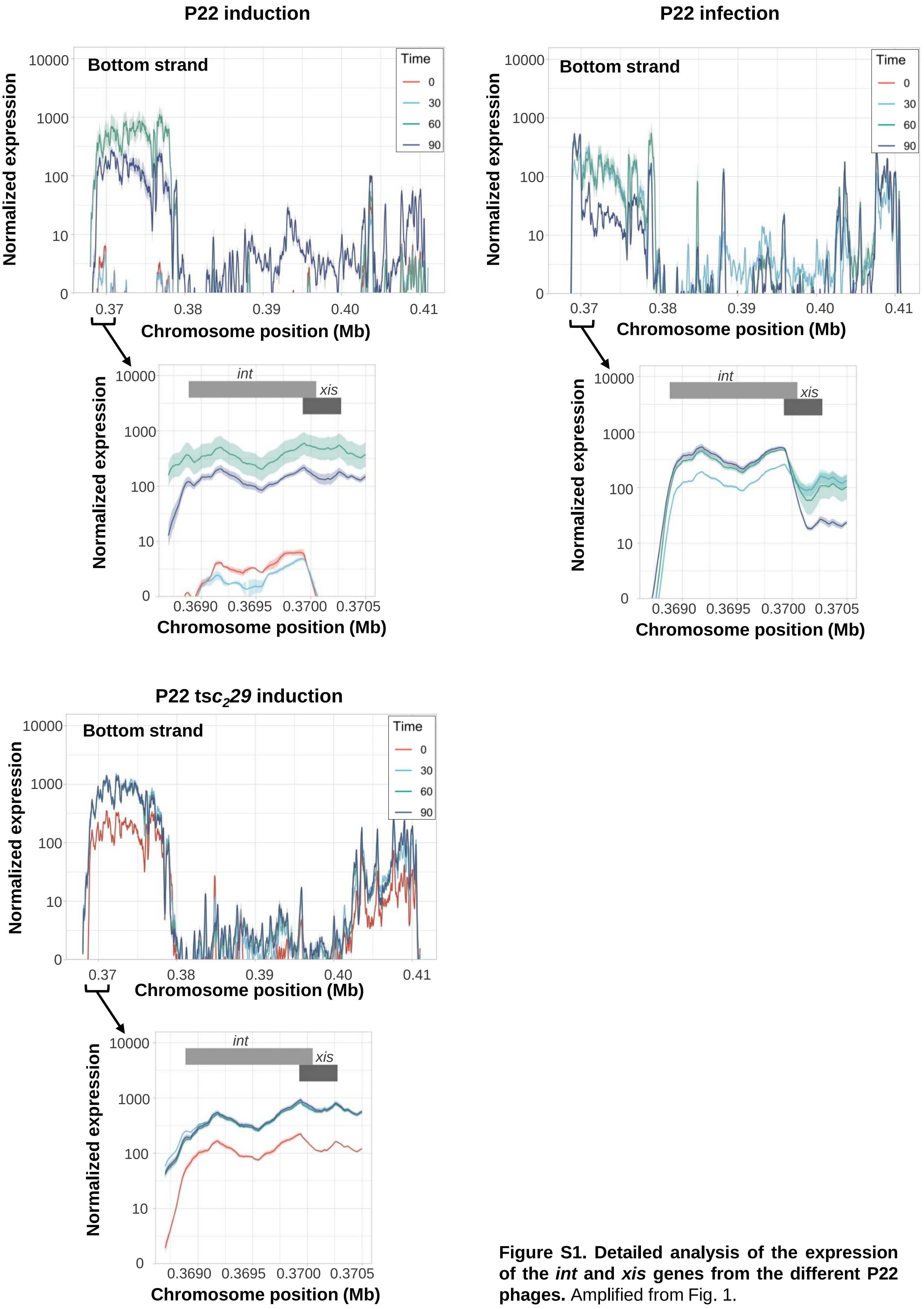

Figure S1. Detailed analysis of the expression of the *int* and *xis* genes from the different P22 phages. Amplified from Fig. 1.

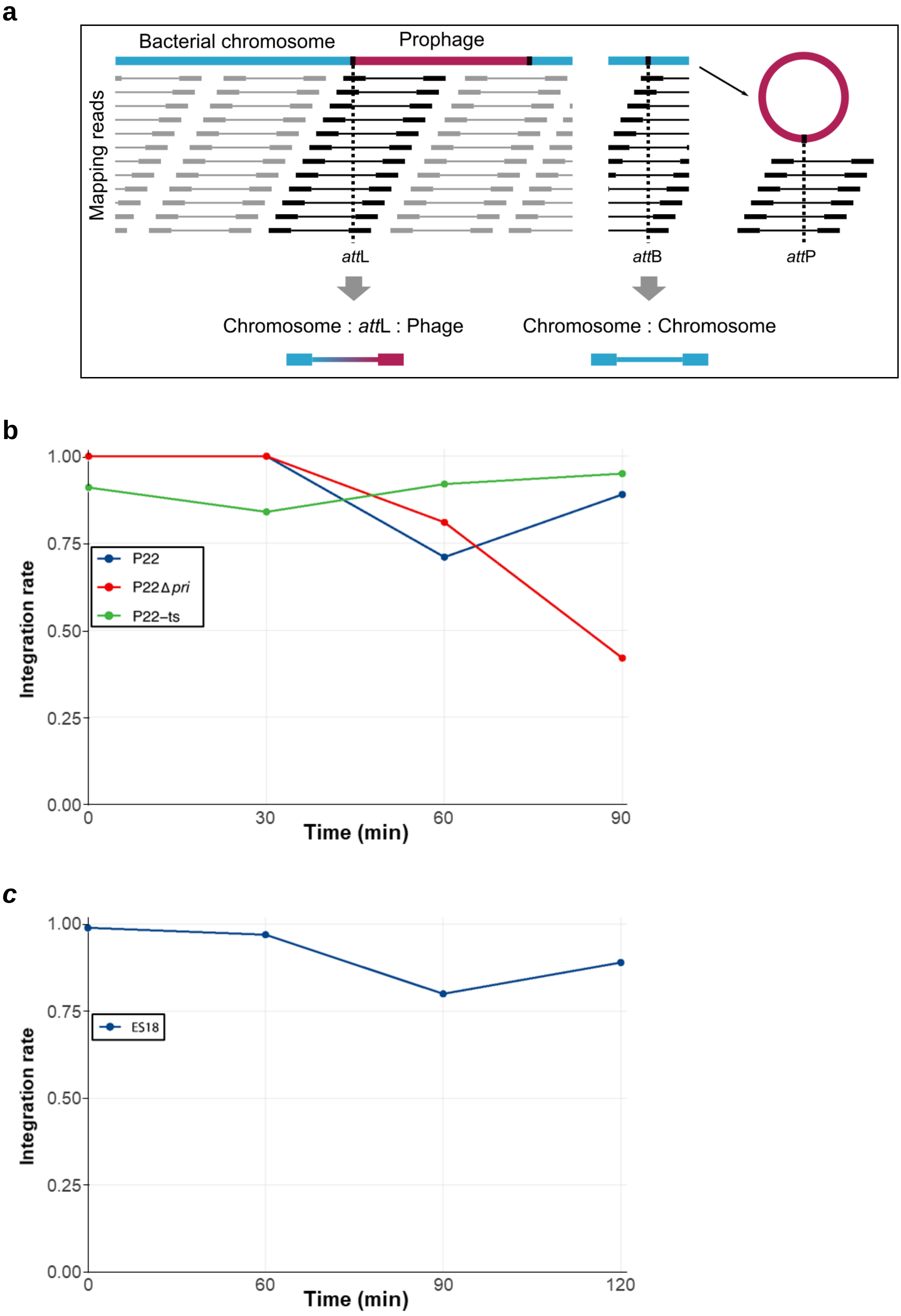

Figure S2. Prophages excise late from the bacterial chromosome.

Schematic representation of sequencing reads used for calculation of integration rate **(a)**, *att*L (left site attachment site) and *att*B (bacterial attachment site). To gain insight into the timing of phage excision, the proportion of integrated phage at each timepoint was calculated by dividing the number of chromosome-*att*L reads by the total number of *att*L/*att*B spanning reads (sum of chromosome-*att*L phage region reads plus reads spanning the *att*B region), obtained from the same sample, for phages P22, P22 Δ*pri* and P22 tsc_2*29* **(b),** and for ES18 **(c)**.

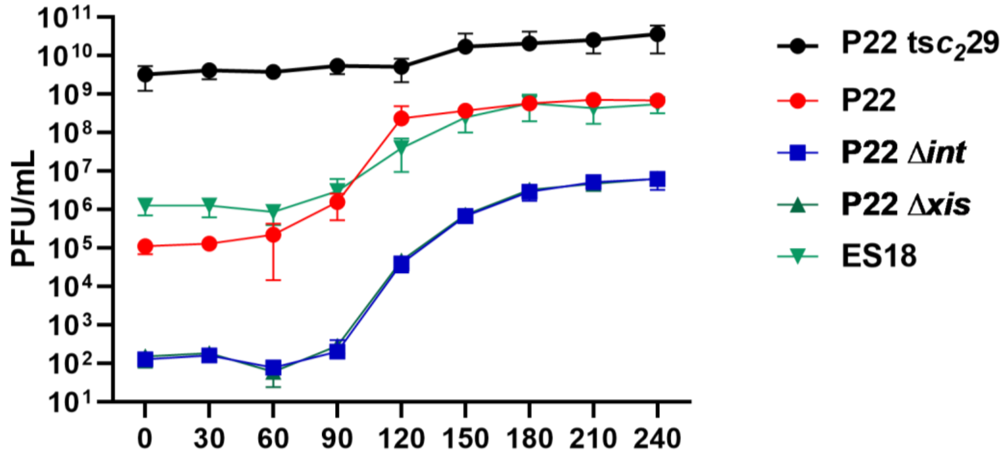

Figure S3. Phage production over a time course experiment after induction.

The lysogenic strains for phage P22, P22 Δ*int*, P22 Δ*xis,* P22 tsc_2*29* and ES18, were mitomycin C or thermal-induced to activate the prophages, samples were taken before (0) and at different time points (every 30 minutes) after induction and the phage titters evaluated (plaque forming units per millilitre: PFU/mL). The means and standard deviations from three independent experiments are presented (*n*=3).

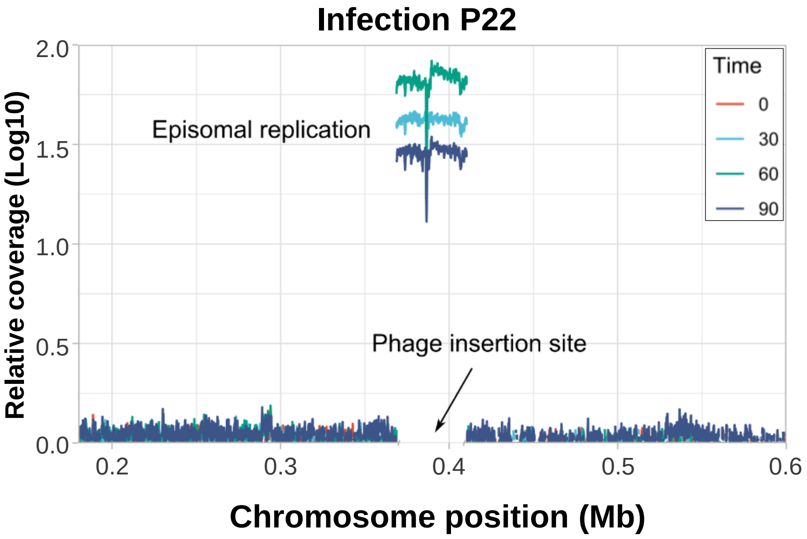

Figure S4. Phage P22 replicates extra-chromosomally after infection.

The relative abundance of phage genomic DNA and the chromosomal regions proximal to where P22 integrates is represented. Samples were analyzed at 0 (red), 30 (cyan), 60 (green), and 90 min (blue) after phage P22 infection. Relative coverage is the DNA relative to the average bacterial genomic coverage (excluding phage).

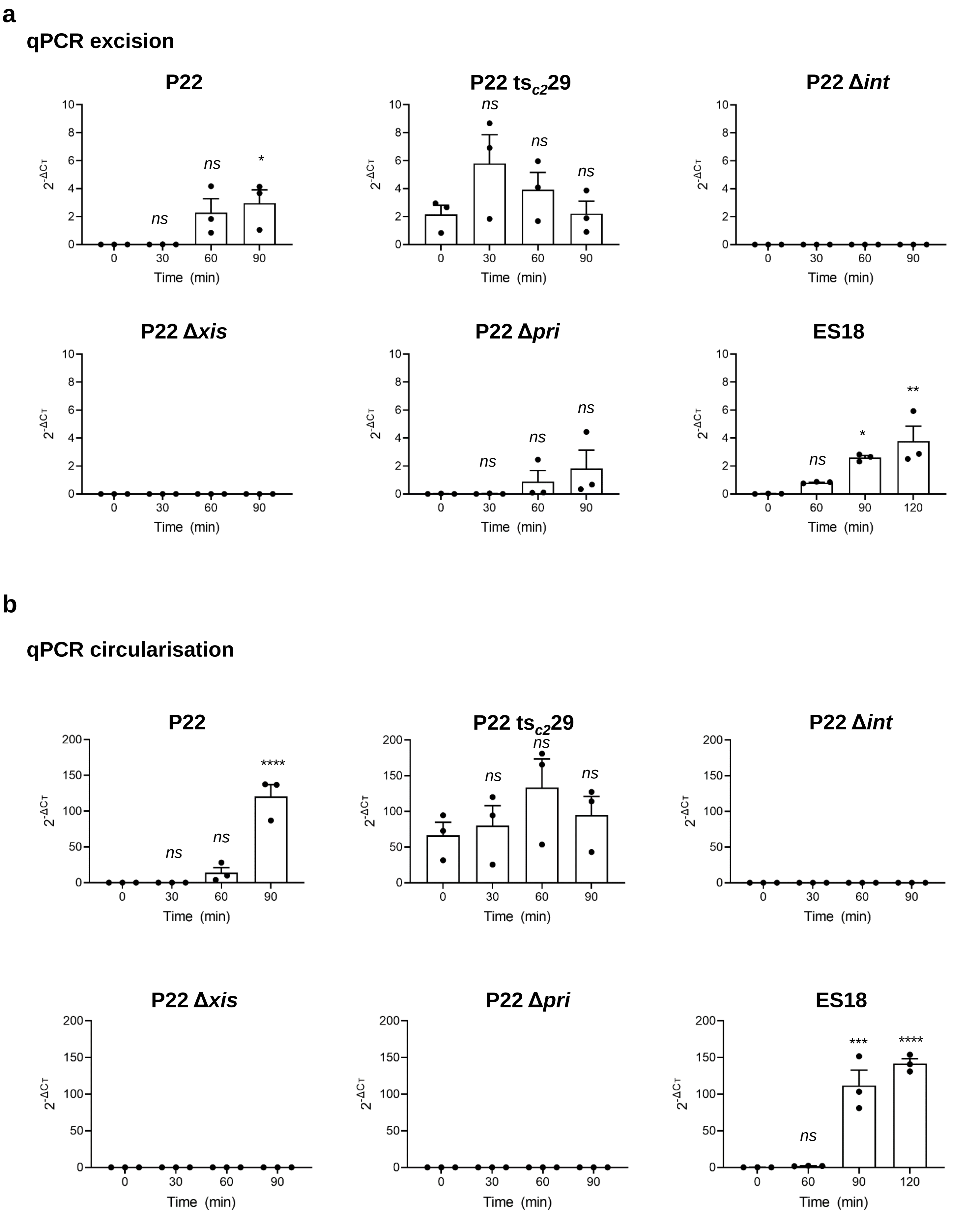

Figure S5. qPCR verification of the strains used in WGS

Analysis of the excision **(a)** or circularisation **(b)** of the different prophages after MC induction, using qPCR. Samples were analysed at 0, 30, 60, and 90 min after induction of the wt or mutants P22 prophages, or at 0, 60, 90, and 120 min after induction of the ES18 prophage (using mitomycin C or temperature-shift, as appropriate). Relative gene expression levels were analyzed by using the $2^{-\Delta CT}$ (where *CT* is threshold cycle). Error bars indicate standard deviation from the mean of three independent experiments. For all panels, values are means (n = 3 independent samples). A one-way ANOVA with Dunnett's multiple comparisons test was performed to compare time 0 against the other timepoints. Adjusted p values were as follows: *ns*>0.05; *p≤0.05; **p≤0.01; ***p≤0.001; ****p≤0.0001. The exact statistical values for each of the conditions tested are listed in Table S1.

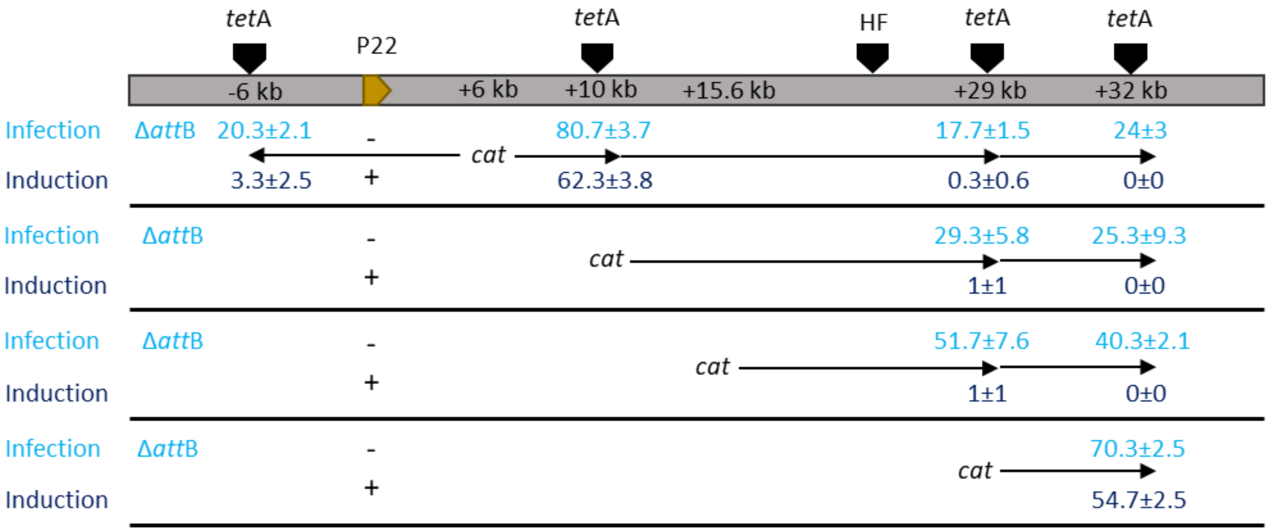

Figure S6. Lateral transduction occurs by the headful mechanism.

Co-transduction frequencies for strains containing both chloramphenicol (*cat)* and tetracycline (*tet*A) markers at varying distances apart. Lysates were tested for co-transduction of both markers by initially selecting for the *tet*A marker, with transductants subsequently scored for the *cat* marker. At least one hundred *tet*A transductants were tested from infection (cyan) or induction (blue) and the results are represented as a percentage (*cat*/*tet*A) x 100%. The *att*B for P22 is highlighted in orange and the headful limit (HF) is indicated. The + indicates a P22 lysogen (induction, LT), while the - indicates a non-lysogenic strain (infection, GT). The means and standard deviations from three independent experiments are presented (*n*=3). Our results indicate that for lysates generated by P22 infection, all co-transduction frequencies were inversely proportional to their distance apart, even if they were located in two different headfuls, indicating that DNA packaging initiated at random sites. However, when we tested lysates generated by SOS induction, co-transduction was only observed for markers within a headful, indicating that packaging had primarily initiated from the bona fide *pac* site, and confirming that P22-mediated LT uses the headful mechanism for packaging.

a

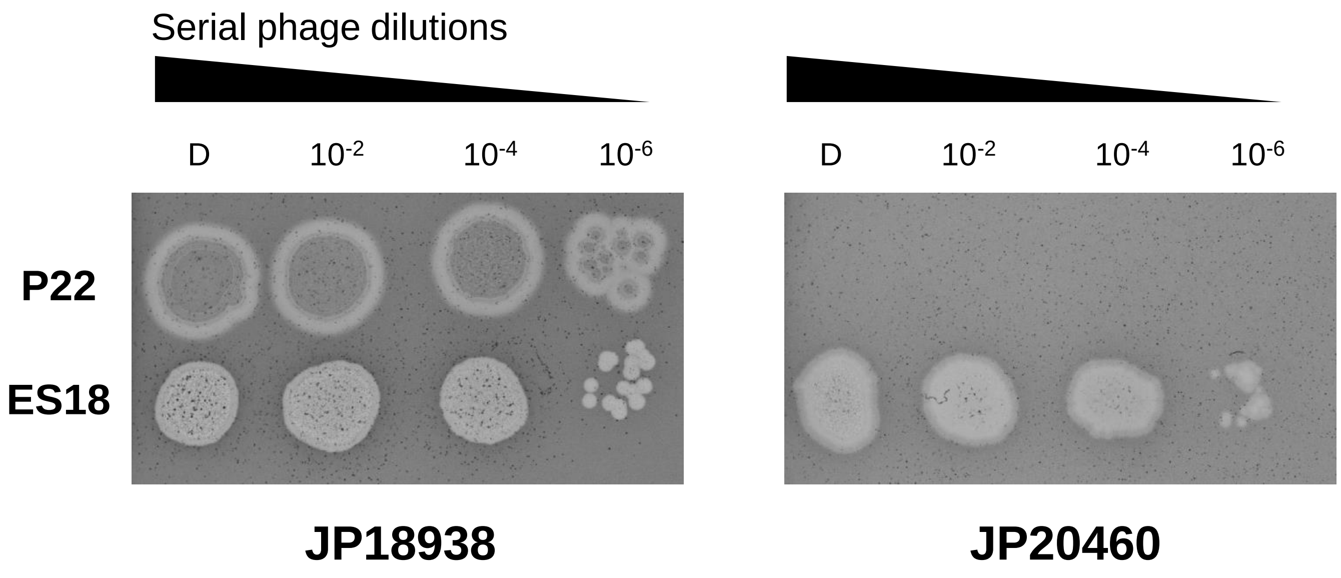

b

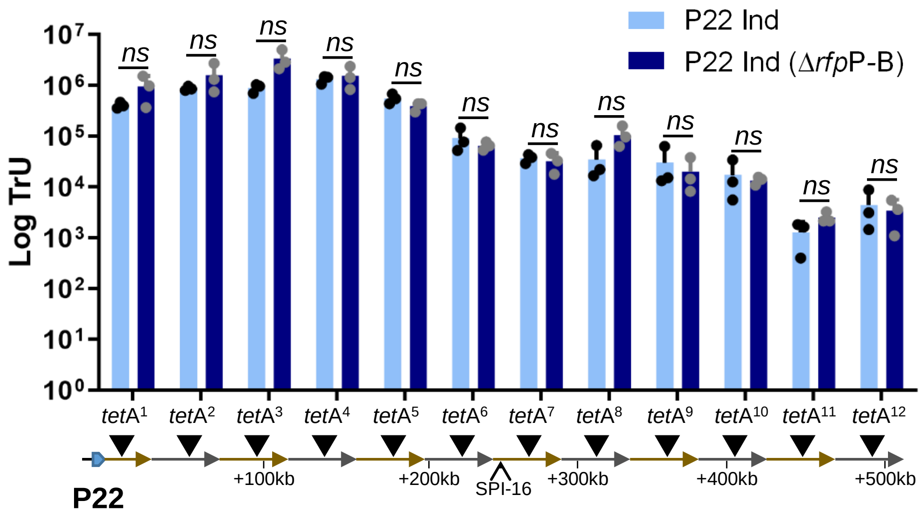

Figure S7. Phage P22 engages in lateral transduction in an infection-resistant host.

To rule out the possibility that P22 phages released early after SOS induction could superinfect the remaining cells to initiate packaging from the resident prophage genome, we deleted the *rfp*P-B genes that encode the P22 receptor and tested this strain for LT. **a,** Deletion of *rfp*PB renders LT2 insensitive to P22 infection. Serial dilutions of phages P22 and ES18 lysates were spotted onto lawns of non-lysogenic LT2 and non-lysogenic LT2 *ΔrfpP-B* strains. The presence of plaques indicates successful phage infection and replication via the lytic cycle. **b,** The transfer of tetracycline (*tet*A) markers located downstream of the P22 *att*B site, in twelve successive capsid headfuls (*tet*A"n"), was tested after P22 induction (Ind) in either wild type LT2 or LT2 lacking the phage P22 receptor (Δ*rfp*P-B). Transduction units (TrU) per milliliter were normalized by PFU per milliliter and represented as the log TrU of an average phage titre (1 x 10^9 PFU). Error bars indicate standard deviation from the mean of three independent experiments. For all panels, values are means (n = 3 independent samples). An unpaired t test two-sided was performed to compare mean differences of infection and induction in each marker. Adjusted p values were as follows: *ns*>0.05; *p≤0.05; **p≤0.01; ***p≤0.001; ****p≤0.0001. The exact statistical values for each of the conditions tested are listed in Table S1.

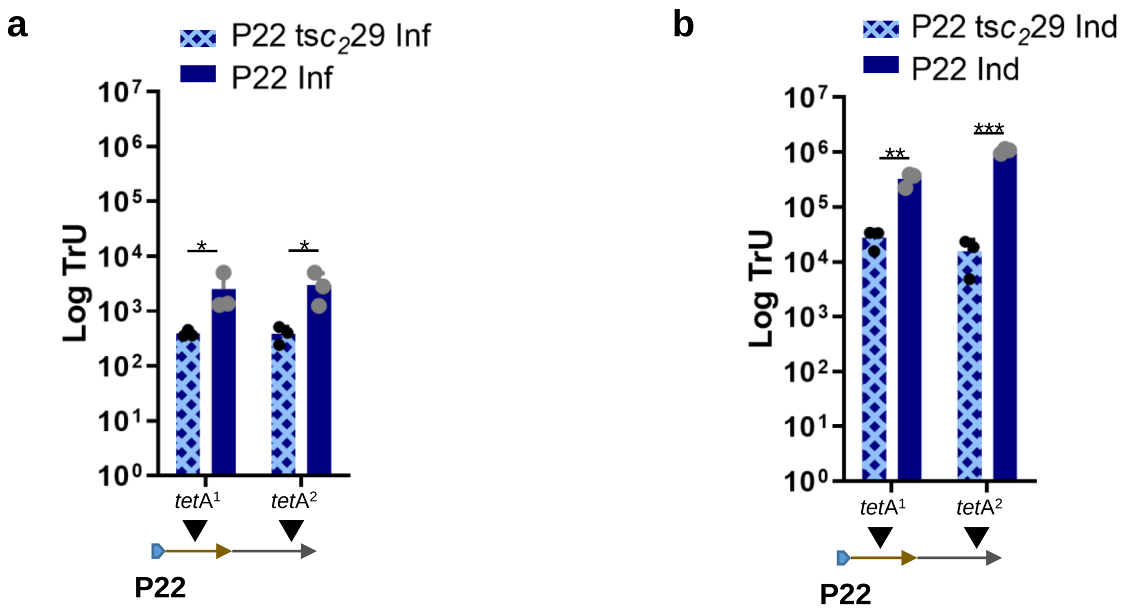

Figure S8. P22 ts$c_2$29 early excision produces low levels of lateral transduction.

a, The transfer by GT of the two first tetracycline (*tet*A) markers located downstream of the P22 *att*B site was compared for both P22 and P22 tsc_2*29* after phage infection (Inf). **b,** The transfer of the two first *tet*A markers located downstream of the P22 *att*B site was compared for both the P22 and the P22 tsc_2*29* prophages following induction (Ind). Transduction units (TrU) per milliliter were normalized by PFU per milliliter and represented as the log TrU of an average phage titre (1 x 10^9 PFU). Error bars indicate standard deviation from the mean of three independent experiments. For all panels, values are means (n = 3 independent samples). An unpaired t test two-sided was performed to compare mean differences of infection and induction in each marker. Adjusted p values were as follows: *ns*>0.05; *p≤0.05; **p≤0.01; ***p≤0.001; ****p≤0.0001. The exact statistical values for each of the conditions tested are listed on Table S1.

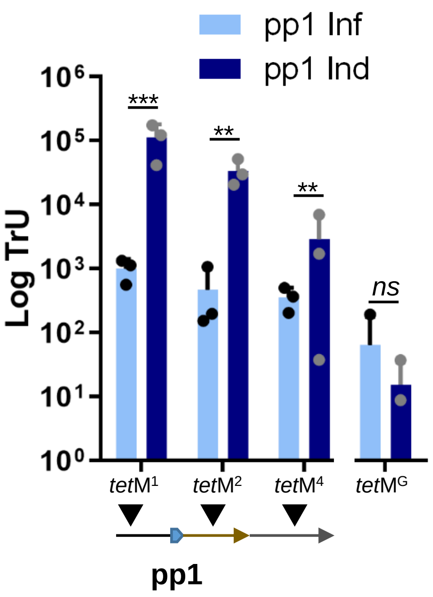

Figure S9. Enterococcal phage pp1 engages in lateral transduction.

The ability of phage pp1 to mobilise tetracycline (*tet*M) markers located downstream of the pp1 *att*B site in successive capsid headfuls (*tet*M$^{\text{"n"}}$), or from non-phage associated regions of the chromosome (*tet*M^{G}), was tested following induction (Ind) and infection (Inf). Transduction units (TrU) per milliliter were normalized by PFU per milliliter and represented as the log TrU of an average phage titre (1 x 10^9 PFU). Error bars indicate standard deviation from the mean of three independent experiments. Values are means (n = 3 independent samples). An unpaired t test two-sided was performed to compare mean differences of infection and induction in each marker. Adjusted p values were as follows: *ns*>0.05; *p≤0.05; **p≤0.01; ***p≤0.001; ****p≤0.0001. The exact statistical values for each of the conditions tested are listed in Table S1.

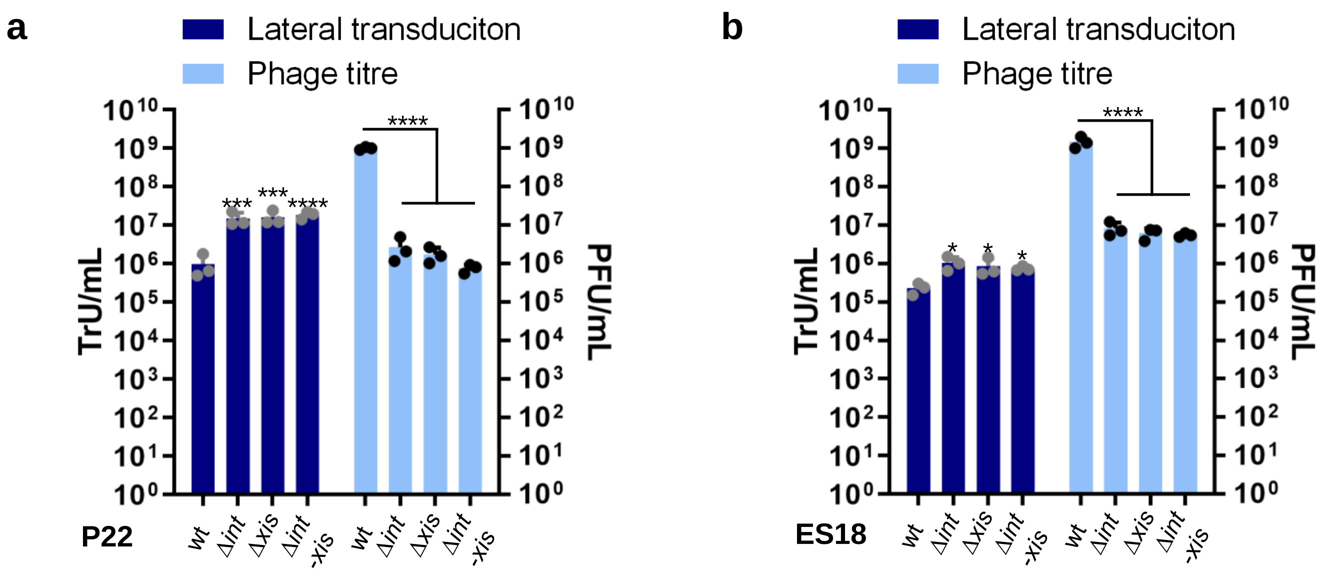

Figure S10. Effect of phage mutations on lateral transduction and phage formation.

a, P22 or P22-derivative (Δ*int*, Δ*xis* or Δ*int-xis*) prophages were mitomycin C induced and tested for their ability to transduce a *tet*A marker (TrU/ml) and produce viable phage particles (PFU/ml). **b,** The same experiment was performed using phage ES18 or ES18-derivates (Δ*int*, Δ*xis* or Δ*int-xis*). The means and standard deviations from three independent experiments are presented (*n*=3). A one-way ANOVA with Dunnett's multiple comparisons test was performed to compare mean differences between time WT and derivatives phages. Adjusted p values were as follows: *ns*>0.05; *p≤0.05; **p≤0.01; ***p≤0.001; ****p≤0.0001. The exact statistical values for each of the conditions tested are listed in Table S1.

References

1. Monk, I. R., Shah, I. M., Xu, M., Tan, M.-W. & Foster, T. J. Transforming the untransformable: application of direct transformation to manipulate genetically *Staphylococcus aureus* and *Staphylococcus epidermidis*. *MBio* **3,** (2012).
2. Matos, R. C. *et al. Enterococcus faecalis* prophage dynamics and contributions to pathogenic traits. *PLoS Genet.* **9,** e1003539 (2013).
3. Datsenko, K. A. & Wanner, B. L. One-step inactivation of chromosomal genes in *Escherichia coli* K-12 using PCR products. *Proc. Natl. Acad. Sci. U.S.A.* **97,** 6640–6645 (2000).
4. Guzman, L. M., Belin, D., Carson, M. J. & Beckwith, J. Tight regulation, modulation, and high-level expression by vectors containing the arabinose PBAD promoter. *J. Bacteriol.* **177,** 4121–4130 (1995).
5. Carpena, N., Manning, K. A., Dokland, T., Marina, A. & Penadés, J. R. Convergent evolution of pathogenicity islands in helper *cos* phage interference. *Philos. Trans. R. Soc. Lond., B, Biol. Sci.* **371,** 20150505 (2016).
6. Charpentier, E. *et al.* Novel cassette-based shuttle vector system for gram-positive bacteria. *Appl. Environ. Microbiol.* **70,** 6076–6085 (2004).
