# Supplementary material for: Lateral transduction is inherent to the life cycle of the archetypical Salmonella phage P22
Source: Nat Commun. 2021 Nov 8;12:6510. doi: 10.1038/s41467-021-26520-4 (PMC8575938; doi:10.1038/s41467-021-26520-4)
Supplement: Supplementary file 3 — Reporting Summary [file 41467_2021_26520_MOESM3_ESM.pdf]

nature research

Corresponding author(s): John Chen
José R Penadés

Last updated by author(s): 22-09-2021

Reporting Summary

Nature Research wishes to improve the reproducibility of the work that we publish. This form provides structure for consistency and transparency in reporting. For further information on Nature Research policies, see our Editorial Policies and the Editorial Policy Checklist.

Statistics

For all statistical analyses, confirm that the following items are present in the figure legend, table legend, main text, or Methods section.

n/a	Confirmed	
☐	☒	The exact sample size (*n*) for each experimental group/condition, given as a discrete number and unit of measurement
☐	☒	A statement on whether measurements were taken from distinct samples or whether the same sample was measured repeatedly
☐	☒	The statistical test(s) used AND whether they are one- or two-sided<br>*Only common tests should be described solely by name; describe more complex techniques in the Methods section.*
☒	☐	A description of all covariates tested
☐	☒	A description of any assumptions or corrections, such as tests of normality and adjustment for multiple comparisons
☐	☒	A full description of the statistical parameters including central tendency (e.g. means) or other basic estimates (e.g. regression coefficient) AND variation (e.g. standard deviation) or associated estimates of uncertainty (e.g. confidence intervals)
☐	☒	For null hypothesis testing, the test statistic (e.g. *F*, *t*, *r*) with confidence intervals, effect sizes, degrees of freedom and *P* value noted<br>*Give P values as exact values whenever suitable.*
☒	☐	For Bayesian analysis, information on the choice of priors and Markov chain Monte Carlo settings
☒	☐	For hierarchical and complex designs, identification of the appropriate level for tests and full reporting of outcomes
☒	☐	Estimates of effect sizes (e.g. Cohen's *d*, Pearson's *r*), indicating how they were calculated

Our web collection on statistics for biologists contains articles on many of the points above.

Software and code

Policy information about availability of computer code

Data collection

No software was used for data collection.

Data analysis

Statistical analyses were performed using GraphPad Prism v6.01 selecting the appropriate transformations and test as indicated in the Materials and Methods as well as in the figure legends within the manuscript.

DNA analyses
FastQC v0.11.8 (http://www.bioinformatics.babraham.ac.uk/projects/fastqc/) was used to assess the quality of the sequencing reads and Trimmomatic v0.3 to remove adapters and low-quality reads. Sequencing reads from each experiment were mapped to their respective reference genomes using the Burrows-Wheeler Alignment Tool 0.7.17 (https://academic.oup.com/bioinformatics/article/25/14/1754/225615). Picard-tools v.2.1.1 (http://broadinstitute.github.io/picard/; Broad Institute) was next used to obtain the bam files, which were merged with SAMtools v1.11, sorted and indexed; and Bedtools bamtobed v2.30.0 was used to produce the bed files. The sequencing reads of the capsids were rarefied by subsampling 100 million reads from each of the paired, filtered reads using seqtk v1.3 sample command (Toolkit for processing sequences in FASTA/Q formats. Available from: http://github.com/lh3/seqtk.). Reads were mapped to the NC_003197 reference genomes and processed as above. The absolute coverage was calculated using bedtools by 100 bp windows across the entire genome. Assemblies of the content of the capsids were obtained using SPAdes v3.1237.

RNA-seq transcriptome analysis
The sequencing reads (Illumina NextSeq 500) were processed using Trimmomatic v0.338 for removal of adapters and low quality reads. Transcriptomic analyses were performed using the pipeline READemption v0.4.3.

For manuscripts utilizing custom algorithms or software that are central to the research but not yet described in published literature, software must be made available to editors and reviewers. We strongly encourage code deposition in a community repository (e.g. GitHub). See the Nature Research guidelines for submitting code & software for further information.

Data

Policy information about availability of data

All manuscripts must include a data availability statement. This statement should provide the following information, where applicable:
- Accession codes, unique identifiers, or web links for publicly available datasets
- A list of figures that have associated raw data
- A description of any restrictions on data availability

Source data are provided with this paper. The WGS and RNAseq data generated in this study have been deposited in the NCBI SRA database under accession code BioProject PRJNA737196 [https://www.ncbi.nlm.nih.gov/bioproject/737196].
The different accession numbers (obtained from the NCBI database) for reference genomes and the bioinformatics analyses are: LT2 (NC_003197), P22 (NC_002371.2) and ES18 (NC_006949).

Field-specific reporting

Please select the one below that is the best fit for your research. If you are not sure, read the appropriate sections before making your selection.

☒ Life sciences ☐ Behavioural & social sciences ☐ Ecological, evolutionary & environmental sciences

For a reference copy of the document with all sections, see nature.com/documents/nr-reporting-summary-flat.pdf

Life sciences study design

All studies must disclose on these points even when the disclosure is negative.

Sample size	No calculations for determining sample size were required for this study. Experiments were repeated three times with defined comparison groups, for example, wild-type against mutant; followed by statistical analysis as described. WGS was performed once and verified by qPCR analysis.
Data exclusions	No data were excluded from the analysis.
Replication	Experiments were performed at least three times as independent biological replicates, which all experimental observations were reproducible for all replication attempts. Information regarding the number of independent replicates performed are included in the methods section of the manuscript, as well as in the figure legends.
Randomization	Randomization was not relevant to this study as phenotypes of strains with clearly defined genetic differences (phage content or gene mutations) were compared.
Blinding	Blinding was not relevant to this study as phenotypes of strains with clearly defined genetic differences (phage content or gene mutations) were compared.

Reporting for specific materials, systems and methods

We require information from authors about some types of materials, experimental systems and methods used in many studies. Here, indicate whether each material, system or method listed is relevant to your study. If you are not sure if a list item applies to your research, read the appropriate section before selecting a response.

Materials & experimental systems

n/a	Involved in the study	
☒	☐	Antibodies
☒	☐	Eukaryotic cell lines
☒	☐	Palaeontology and archaeology
☒	☐	Animals and other organisms
☒	☐	Human research participants
☒	☐	Clinical data
☒	☐	Dual use research of concern

Methods

n/a	Involved in the study	
☒	☐	ChIP-seq
☒	☐	Flow cytometry
☒	☐	MRI-based neuroimaging
